# Reflections on a journey as sleep researcher and geriatric psychiatrist

**DOI:** 10.1093/sleepadvances/zpad032

**Published:** 2023-11-08

**Authors:** Charles F Reynolds

**Affiliations:** Distinguished Professor of Psychiatry and UPMC Endowed Professor in Geriatric Psychiatry, Emeritus, University of Pittsburgh School of Medicine, Pittsburgh, Pennsylvania 15213, USA

**Keywords:** sleep, aging, mental illness

## Abstract

After first recalling the origins of my interest in sleep and dreams at UVa (1969) and my MD thesis at Yale on sleep in mood disorders (1973), I will describe my service to the field of sleep disorders medicine, through various roles in the American Sleep Disorders Association, the Institute of Medicine, the National Institute of Mental Health, and the DSM-5 Task Force of the American Psychiatric Association. I will then present the broad themes of my contributions to psychiatric sleep research, focusing on the neurobiology of sleep as a dimension of the risk and protective architecture for depression in older adults, as a bridge to diagnostic and treatment issues in later-life depression, and to clinical and translational neuroscience addressing the intersections of sleep, aging, and mind/brain health. Throughout this narrative, I highlight many relationships with mentors and mentees. All of my scientific activity has been team-based, providing the social matrix for the physician–scientist I have become.

This paper is part of the Living Legends in Sleep Research series, which is sponsored by Idorsia Pharmaceuticals and Jazz Pharmaceuticals.

## Who have I become?

My academic title is Distinguished Professor of Psychiatry and UPMC Endowed Professor in Geriatric Psychiatry *(emeritus)* at the University of Pittsburgh School of Medicine. Before becoming *emeritus* on July 1, 2017, I also served as Professor of Behavioral and Community Health Science at the University of Pittsburgh Graduate School of Public Health, in addition to serving as the Director of the Aging Institute of UPMC Senior Services and the University of Pittsburgh (www.aging.upmc.com). I directed an NIMH-sponsored P30 Advanced Center for Interventions and Services Research in the Prevention and Treatment of Late-Life Mood Disorders (1995–2017) and the John A. Hartford Foundation Center of Excellence in Geriatric Psychiatry (2005–2017). The themes of my research program have encompassed (1) prevention and treatment of depression and its complications in older adults (in high-, low-, and middle-income countries), (2) understanding treatment response variability from neurobiological (including sleep) and psychosocial perspectives and applying that understanding to the development and testing of new ways to manage difficult-to-treat depression in later life, and (3) devising and testing models of improved mental health services delivery for older disadvantaged (often minority) adults living with mood disorders. In addition, I have collaborated in research on the diagnosis and treatment of prolonged grief disorder (now incorporated into the DSM 5 TR). As elaborated below, sleep research has provided a conceptual basis and an empirical bridge to all of these themes. My CV now includes over 820 peer-reviewed publications and 185 invited manuscripts, with 65,309 citations, and an H-Index of 109 in June, 2023, as calculated using *Clarivate/Web of Science*. I have also delivered over 200 invited platform presentations, many dealing with psychiatric sleep research, in the United States, Europe, Canada, Japan, Australia, New Zealand, Brazil, and India. My track record as a physician–scientist has rested upon a foundation of global interdisciplinary collaboration for over four decades. Since 2016, I have served editor-in-chief of the *American Journal of Geriatric Psychiatry*—an ideal platform for continuing to build the field and to nourish the careers of those who will someday become its leaders (including colleagues with specific interests in sleep, aging, and mental illness).

## Paying it forward

Mentoring early career scientists has been an important focus of my career as a physician scientist (and an obligation of having received K awards for 20 years). In this context, I have served as senior advisor to the NIMH-sponsored Advanced Research Institute in Geriatric Mental Health since its inception. As previous director of both institutional training grants (T32’s) and psychiatric research education grants (R25’s), I have mentored over 25 K-awardees, the majority of whom have competed successfully for R01 grants. In recognition of my commitment to mentoring, I was the 2022 recipient of the Julius Axelrod award for excellence in mentoring, presented by the American College of Neuropsychopharmacology (ACNP). I recently served as co-Chair of the planning committee for a workshop at the National Academy of Science, Engineer, and Medicine, addressing the rising mental health needs of older adults (May 15–16, 2023). My work as a clinical investigator has been recognized by the American Psychiatric Association (through conferral of the Jack Weinberg Award in 2012 for lifetime achievement in geriatric psychiatry), the Brain and Behavior Research Foundation (through conferral of the 2016 Herbert Pardes Humanitarian Prize) and by the American Society for Clinical Psychopharmacology (through conferral of the 2022 Donald Klein Lifetime Achievement Award). I want also to celebrate the achievements of colleagues I’ve mentored, giving a shout-out especially to Dan Buysse MD, Eric Nofzinger MD, the late Tica Hall PHD, and the late Carolyn Hoch PhD.

## Origins of my interest in sleep and dreams

I majored in philosophy and religious studies as an Echols scholar at the University of Virginia, where my mentor was Professor David Harned, Ph.D., a scholar transplanted from Yale. David (who was also a minister in the Lutheran church) encouraged my interest in scholarly activities and in the humanities. In addition, UVa faculty transplanted from Oxford guided my studies of epistemology and moral philosophy, which took the form of weekly tutorial readings and essay writing. Learning to read and to write carefully prepared me well for a career in academic medicine. I read the works of Sigmund Freud, particularly his introductory lectures on psychoanalysis, his study of civilization and its discontents, and his work in dreams and the psychopathology of everyday life. I felt a strong calling to become a physician, with a particular interest in academic psychiatry. This was what attracted me to Yale, with its world-renowned Department of Psychiatry. Professor Harned encouraged me to apply to Yale, recognizing in me a strong self-guided learner and perceiving an excellent fit for my interests and temperament with Yale medicine. He was indeed correct. I spent many hours in the Cushing Medical Library, reading literature in psychoanalysis, while also immersing myself in neurology, internal medicine, and in the burgeoning field of biological psychiatry, in which I benefitted from being mentored by David Kupfer MD and Thomas Detre MD. David’s work in sleep and mood disorders, and his discovery of shortened REM sleep latency in depression, held great excitement for me. Although I also had a Danforth Foundation graduate fellowship to pursue a PhD in religious studies at Yale, I did not take this opportunity but chose instead to immerse myself in psychiatric sleep research and to pursue a career in academic psychiatry. However, my interest in philosophy and religious studies continues to this day and strengthens the humanistic dimension of my clinical and scientific activities.

## MD thesis at Yale

As a third- and fourth-year medical student, I pursued my MD thesis research on the clinical research unit of the Connecticut Mental Health Center. Taking advantage of an extant data set of EEG sleep recordings in inpatients with bipolar disorder, before and during treatment with lithium carbonate. I observed that administration of lithium led to improvement in sleep health, and particularly to a restoration of slow-wave sleep correlating with both steady-state lithium plasma levels and with clinical improvement.

Yale celebrated my thesis on medical student research day in 1973. David and I published data from the thesis in the *Archives of General Psychiatry*—a very heady experience for me (please see citation [1]).

My thesis also included a literature review of maintenance pharmacotherapy in bipolar disorder—presaging my interest in sleep pathophysiology as a bridge to psychiatric diagnosis, and to treatment response variability during acute, continuation, and maintenance therapy of severe mood disorders in older adults. The work with David and its success as a peer-reviewed paper in the field’s top journal reinforced my interest in academic psychiatry and in the marriage of sleep and intervention research. I accepted an invitation from David and from Thomas Detre MD to follow them to Pittsburgh for my psychiatry residency and then to remain on faculty (1977–2017). Following graduation from Yale, I took an interim year (1973–1974) to do a straight medical internship at McGill, under Professor John Beck MD, a leader in the nascent field of geriatric medicine, whom I’d met during an infectious disease rotation in 1971 at the Kenyatta National Hospital in Nairobi. This experience in Nairobi contributed to the genesis of my interest in global health. My time in Montreal included rotations at the Allan Memorial Institute in psychiatry and at the Montreal Neurological Hospital, where I met and had dinner with Professor Wilder Penfield. From Yale, to Nairobi and Montreal, and finally to Pittsburgh—a circuitous route, yes, but, as it turned out, a thematically focused journey lasting four decades and grounded in psychiatric sleep research.

## Becoming a geriatric psychiatrist and sleep researcher: defining the foundational theme of my work

While serving as an assistant professor of psychiatry at Pitt, David and Thomas encouraged me to prepare an NIH research career development (K) award, addressing my interests in the sleep of older adults living with mood disorders. This was for me a compelling extension of my MD thesis work at Yale. My K01 application (entitled “Sleep, Aging, and Mental Illness”) garnered a perfect score of 100, following a site visit conducted by Professor Jerry Vogel, a sleep scientist and psychiatrist whose work in the antidepressant effects of REM sleep deprivation I much admired. The K01 would be the start of a 20-year journey (1980–2000) as a K awardee (K01, K02, and K05- the last a senior research scientist award from NIMH), both researching and mentoring in the intersecting fields of sleep research and geriatric psychiatry, while also building a successful program as principal investigator through research project grants (RO1’s) and center grants (P30’s) from NIMH totaling over $77,000,000. During this journey I benefitted from being mentored by Barry Lebowitz at NIMH, and by illustrious sleep scientists—David Kupfer, Alan Rechtschaffen, Bill Dement, Christian Guilleminault, Jerry Vogel, and Rosalind Cartwright. These friendships confirmed and deepened my commitment to rendering service and pursuing sleep research, as an early career citizen in the burgeoning field of sleep disorders medicine. I never strayed from my fascination with the sleeping brain as a physiological window into understanding differential diagnosis and treatment response variability among older adults with mood disorders. More specifically, my science has addressed sleep as a window into the health of the aging brain, as a marker of vulnerability to incident and recurrent episodes of major depression, and as a reflection of cognitive reserve and vulnerability to cognitive decline. Sleep changes also provide a window into the effects of major life stressors, such as bereavement, on mind/brain health, and how such effects differ from those associated with depression and dementia.

## Bridging service and science in the field of sleep disorders medicine

While pursuing my K-sponsored research in sleep, aging, and mental illness, I was inspired by Bill Dement, Howard Roffwarg, Christian Guilleminault, John Karacan, and Helio Lemmi, among others, to become active in the American Sleep Disorders Association (ASDA) and to get certified as a sleep disorders specialist by the American Board of Sleep Disorders Medicine (1991). In doing so I felt welcome and was excited to become part of a new community of physician scientists. I served as a member of three ASDA committees, addressing certification, standardization, and education, and in that capacity chaired many site visits to clinical sleep laboratories around the United States. I also chaired the Nathaniel Kleitman Distinguished Service Award Committee, where I was inspired by colleagues who had grown great in service to our field. My own service to the field of sleep disorders medicine and science would eventually also include participation in an Institute of Medicine (IOM) study of sleep disorders and their public health burden (2005–2006), followed by chairmanship of the DSM-5 workgroup on sleep–wake disorders, in collaboration with my Stanford colleague and friend, Professor Ruth O’Hara (2006–2013). The DSM-5 nosology of sleep–wake disorders (2013) represented an important scientific and clinical advance over its predecessors in DSM-III and DSM-IV (to both of which I had also contributed). Unlike all other disorders in the DSM-5, we included in the diagnostic criteria objective measures and biomarkers, derived from sleep laboratory-based research in insomnia disorders, disorders of excessive sleepiness, breathing-related sleep disorders, parasomnias, and circadian rhythm disorders. This work provided further and broader context, both clinical and scientific, for my studies of sleep in normal aging, depression, and dementia. In my capacity as work-group chair, I authored and co-authored several manuscripts describing the scientific basis of the DSM-5 sleep wake disorders nosology, in collaboration with Maurice Ohayon at Stanford (please see citations [2–4]). Throughout all of this activity, I served on initial review groups (study sections) at the National Institute of Mental Health (NIMH), chairing the Psychopathology and Clinical Biology study section, and on the National Mental Health Advisory Council (2003–2006).

## Conceptual Foundation and themes of my research in sleep, aging, and mental illness

As a K01 recipient (1980–1985), I competed successfully in 1983 for my first R01 from the NIMH dealing with sleep, aging, and mental illness—an R01 which I held for 25 years, through several successful competing continuations. The focus of this work gradually evolved from issues related to diagnosis and course of illness to treatment-related issues, informed by my clinical practice as a geriatric psychiatrists. This R01 provided a bridge to my interests in the sleep-related pathophysiological basis of illness vulnerability and course, as well as differential diagnosis and treatment response variability of mood disorders in older adults, including bereavement-related depression and prolonged grief disorder.

The conceptual foundation of my sleep and aging research is the view that sleep provides a unique and powerful window into brain health—as expressed in cognitive and affective fitness in later life. Alex Borbely’s two-process model of sleep–wake regulation (homeostatic and circadian) provided a useful theoretical framework for my descriptive EEG sleep studies of depression and dementia in old age. In a series of papers published in the *Archives of General Psychiatry (*the predecessor of JAMA Psychiatry and the most prestigious journal in psychiatric research), my research team described both the macro- and microarchitecture of EEG sleep in late-life major depression and dementia, as contrasted with the sleep of healthy aged adults [5–19]. We observed differences in both baseline sleep architecture and following the use of various probes, such as total sleep deprivation and REM sleep deprivation. For example, we observed not only preservation but also increases in tonic and phasic REM sleep and REM activity in depression, in contrast to the loss of REM sleep time, activity, and density in persons with dementia. The temporal distribution of REM sleep and of rapid eye movement activity also differed in depression from that seen in normal sleep and in the sleep of demented patients. These differences in REM sleep macro- and micro-architecture co-occurred with reduced slow wave sleep activity (as assessed through period and amplitude analysis), especially in the first non-REM sleep period. In response to sleep deprivation as a probe, older depressed patients were able to generate a slow wave sleep rebound, attesting to restoration of homeostatic regulation, while such capacity was lacking in older demented patients. Older adults with dementia showed not only diminished slow wave activity (in period and amplitude analysis) but also decreased K complexes and spindles during NREM sleep. Similarly, in response to REM sleep deprivation, REM rebound was evident in depression but not in dementia. These data were consistent with the sleep-cognition hypothesis, which posited a deep, reciprocal relationship between cognitive health and sleep health. Dan Buysse and I extended this hypothesis into longitudinal EEG sleep studies of patients with so-called “depressive pseudodementia,” in which we observed preservation of REM sleep in depressed elderly with reversible cognitive impairment. Carolyn Hoch and I also published observations of increased rates of sleep-disordered breathing in older adults with dementia. Ultimately, in work led by Eric Nofzinger, we integrated sleep research with the nascent field of brain imaging studies, using positron emission tomography (PET), to visualize brain activity during transitions to REM sleep in persons with depression. In work led by Macheri Keshavan, we used P31 NMR spectroscopy to understand the bioenergetic correlates of diminished slow wave sleep in psychotic disorders. In collaboration with Michael Thase, Ellen Frank, and Eric Nofzinger, my team carried out studies of nocturnal penile tumescence in depressed men before and after treatment with cognitive behavioral therapy.

One particular project of our team’s work, with probably the greatest subsequent impact on research and clinical practice, featured the development and performance characteristics of the Pittsburgh Sleep Quality Index (PSQI), now translated into over 56 languages and used world-wide [19].

## Marrying psychiatric sleep research with intervention science (treatment and prevention)

My R01 activity extended gradually beyond describing EEG sleep characteristics of later-life mood and cognitive disorders to embrace both bereavement-related depression and an examination of the effects on sleep of depression-specific pharmacotherapy and psychotherapy (both Interpersonal Psychotherapy and Cognitive Behavioral Therapy). These data were collected in the context of acute, continuation, and maintenance treatment research funded by NIMH (please see citations [20–27]).

My team’s studies of sleep as a correlate of treatment response variability in later-life depression led in 1989 to award of a 10-year MERIT award (R37) from the NIMH, to support long-term, randomized controlled trials (RCTs) of maintenance treatment in older adults with major depression. My program officer Dr. Barry Leibowitz and the Director of NIMH, Dr. Lew Judd, were very supportive of my research. Drs. David Kupfer, Ellen Frank, and Jim Perel were my dedicated collaborators and friends in this work. Over a 20-year period, my team published three double-blind, placebo-controlled RCTs addressing the efficacy of pharmacotherapy and psychotherapy in either preventing recurrent episodes of major depression or delaying onset of dementia [28–30].

My interest in the prevention of recurrent episodes of major depression ultimately broadened to encompass prevention of incident (first) episodes of major depression in vulnerable older adults, which I came to see as a global priority during a visiting professorship at the Free University of Amsterdam with Professors Pim Cuijpers and Aartjan Beekman [31].

In collaboration with Professors Vikram Patel, Pim Cuijpers, and other colleagues, I returned to my interest in global mental health and to the use of a brief behavioral treatment for insomnia (BBTI, developed and tested for efficacy by Dan Buysse in primary care older adults), combined with problem solving therapy for primary care (PST-PC), as strategies for use in the indicated prevention of major depression. Vikram, Pim, and I competed successfully for an intervention development grant from NIMH, to study the efficacy of a simple behavioral intervention, deliverable by lay counselors, for preventing incident major depression in older adults attending rural and urban primary care clinics in Goa, India. Compared with care as usual, our model combining PST-PC and BBTI proved efficacious in protecting older adults with subsyndromal symptoms of depression from transitioning to episodes of frank major depression (an example of “indicated” depression prevention, in the Institute of Medicine lexicon), while also conferring benefits on blood pressure control [32].

## Activities as an *Emeritus* Professor: 2017–present

My professional commitments now embrace editorship of the *American Journal of Geriatric Psychiatry* (since 2016), membership on the editorial board of *JAMA Psychiatry*, chairmanship of a recent workshop at the National Academy of Science, Engineering, and Medicine (May 15-15, 2023: “Addressing the Rising Mental Health Needs of an Aging Population”), mentoring early career scientists in sleep and geriatric psychiatry in their K and R awards, and continuing publication of opinion pieces in *JAMA Psychiatry* and manuscripts in the *Lancet* and in *World Psychiatry*, in collaboration with a global network of colleagues [33–36].

## Conclusion

As I reflect on the journey that my spouse Ellen and I continue to enjoy, blessed as we are with good health, I am reminded that our present is never very far from our past or our future (and that, as T. S. Eliot wrote in Four Quartets, it is often in re-visiting a place or recalling an activity that we then and only then know them for the first time). I am grateful to my colleagues, mentors, and mentees in sleep research for the starting point and indeed the long and happy continuation of my journey. The memory of these relationships remains, and will remain, a blessing for the rest of my life. As one of my favorite poets, Mary Oliver, has written in *The Gift*:

Be still, my soul, and steadfast.
Earth and heaven both are still watching
though time is draining from the clock
and your walk, that was confident and quick,
has become slower.

So, be slow if you must, but let
the heart still plays its true part.
Love still, as once you loved, deeply
and without patience. Let God and the world
know you are grateful.
That the gift has been given.
